# Calcium oxalate toxicity in renal epithelial cells: the mediation of crystal size on cell death mode

**DOI:** 10.1038/cddiscovery.2015.55

**Published:** 2015-11-23

**Authors:** X-Y Sun, Q-Z Gan, J-M Ouyang

**Affiliations:** 1 Department of Chemistry, Jinan University, Guangzhou 510632, China; 2 Institute of Biomineralization and Lithiasis Research, Guangzhou 510632, China

## Abstract

The cytotoxicity of calcium oxalate (CaOx) in renal epithelial cells has been studied extensively, but the cell death mode induced by CaOx with different physical properties, such as crystal size and crystal phase, has not been studied in detail. In this study, we comparatively investigated the differences of cell death mode induced by nano-sized (50 nm) and micron-sized (10 *μ*m) calcium oxalate monohydrate (COM) and calcium oxalate dihydrate (COD) to explore the cell death mechanism. The effect of the exposure of nano-/micron-sized COM and COD crystals toward the African green monkey renal epithelial (Vero) cells were investigated by detecting cell cytoskeleton changes, lysosomal integrity, mitochondrial membrane potential (Δ*ψ*m), apoptosis and/or necrosis, osteopontin (OPN) expression, and malondialdehyde (MDA) release. Nano-/micron-sized COM and COD crystals could cause apoptosis and necrosis simultaneously. Nano-sized crystals primarily caused apoptotic cell death, leading to cell shrinkage, phosphatidylserine ectropion, and nuclear shrinkage, whereas micron-sized crystals primarily caused necrotic cell death, leading to cell swelling and cell membrane and lysosome rupture. Nano-sized COM and COD crystals induced much greater cell death (sum of apoptosis and necrosis) than micron-sized crystals, and COM crystals showed higher cytotoxicity than the same-sized COD crystals. Both apoptosis and necrosis could lead to mitochondria depolarization and elevate the expression of OPN and the generation of lipid peroxidation product MDA. The amount of expressed OPN and generated MDA was positively related to cell injury degree. The physicochemical properties of crystals could affect the cell death mode. The results of this study may provide a basis for future studies on cell death mechanisms.

## Introduction

More than 70% of renal stone patients suffer from urolithiasis caused by calcium oxalate (CaOx) stones, of which calcium oxalate dihydrate (COD) is the most common crystal in healthy human urine, and calcium oxalate monohydrate (COM) is the most common crystal in renal stones.^1,2^ In the absence of medical treatment, nephrolithiasis is a recurrent disease, with a prevalence of 50% over 10 years.^1^

Many researchers have recently emphasized that the interaction between crystals and renal tubular epithelial cells, including the adhesion or endocytosis of crystals by cells, is an important factor in stone formation.^3,4^ These processes could lead to cellular injury, alterations in cellular structure, compositions, physiology and gene expression, initiation of DNA synthesis, and ultimately cell death.^5,6^

Although cell death caused by CaOx crystals has been extensively studied, the mode of cell death produced by CaOx has not been defined. Many researchers have demonstrated that exposure of cells to CaOx crystals can lead to significant apoptotic changes, including condensation and margination of nuclear chromatin, DNA fragmentation, and migration of phosphatidylserine (PS) of the plasma membrane from inside the cell membrane to the cell surface.^5,7^ However, other researchers have also proven that exposure of cells to CaOx crystals results in necrotic cell death with significant necrotic changes, such as loss of plasma membrane integrity, release of lactate dehydrogenase, cellular and nuclear swelling, and inflammatory response.^8–10^ Furthermore, CaOx exposure can also simultaneously induce both apoptotic and necrotic cell death.^11^ Apoptosis has come to be used synonymously with the phrase ‘programmed cell death’ as it is a cell intrinsic mechanism for suicide that is regulated by a variety of cellular signaling pathways. In contrast to apoptosis, necrosis has been traditionally thought to be a passive form of cell death with more similarities to a train wreck than a suicide.^12,13^ In general, fast-acting metabolic poisons and strong physical stress, such as freezing, boiling, or shearing, rupture cell membranes and cause rapid cell necrosis. By contrast, a slow-acting form of cell death called apoptosis does not involve membrane damage and inflammation.^14,15^ Therefore, cell death is a complicated pathological process. Cell apoptosis and necrosis caused by CaOx crystal exposure may be related to cell types, crystal concentration, exposure time, and even the unknown physicochemical properties of crystals.

The urine of normal and kidney stone patients all contained numerous of COM and COD crystals, these crystals often had different sizes ranged from a few nanometers to tens of microns.^16^ In our early study,^17^ we analyzed the crystalline size in the urine samples of 85 healthy persons and 65 lithogenic patients, most of the nanocrystallites in healthy urine samples were with a narrow particle size distribution from about 20 to 400 nm, but most of the particles in lithogenic urines had a broad particle size distribution from 1.1 to 1000 nm. The size of exogenous particles affects their cytotoxicity,^18,19^ but the mode of cell death caused by nano-/micron-sized crystals has not been studied. Thus, the present study focused on the cell death mode by apoptosis or necrosis induced by nano-/micron-sized COM and COD crystals to reveal their cell death mechanism.

## Results

### Characterization of nano-/micron-sized COM and COD

**Figure 1:**
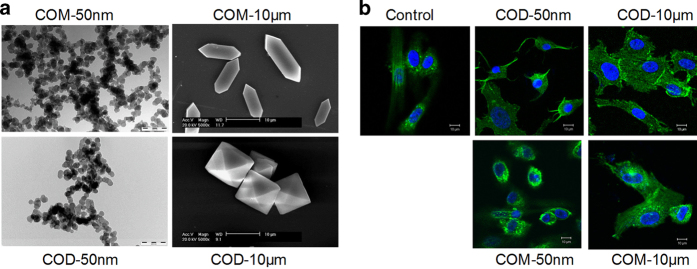
Morphological observation of nano-/micron-sized COM and COD crystals and cytoskeleton. (**a**) SEM and TEM images of nano-/micron-sized COM and COD crystals, respectively. (**b**) Confocal laser scanning microscopy observation of the changes of cytoskeleton caused by nano-/micron-sized COM and COD crystals. Cell nuclei (blue) and cytoskeleton (green, as represented by F-actin) were stained with DAPI and FITC-conjugated phalloidin, respectively. Crystal concentration: 200 *μ*g/ml.

Figure 1a shows the transmission electron microscope (TEM) images of the nano-sized COM and COD crystals, and scanning electron microscope (SEM) images of the micron-sized COM and COD crystals. Their sizes are almost 50 nm and 10 *μ*m, respectively. We used an integer (COM-50 nm, COM-10 *μ*m, COD-50 nm, and COD-10 *μ*m) to represent the crystal size for simplicity and convenience.

COM-50 nm and COD-50 nm were nearly spherical, COM-10 *μ*m transformed into hexagonal lozenge, whereas COD-10 *μ*m transformed into tetragonal bipyramid. The crystal phase was detected by XRD and FT-IR characterization in our present study,^20^ all the prepared samples were pure-phase COM crystals or COD crystals.

### Cell cytoskeleton changes

For the exploration of the difference of cell death mode caused by different-sized CaOx crystals, African green monkey renal epithelial (Vero) cells were co-cultured with 200 *μ*g/ml nano-/micron-sized COM and COD crystals for 6 h. Actin filaments are important protein fibers of the cytoskeleton. Thus, we stained F-actin filaments with fluorescein isothiocyanate (FITC)-phalloidin to observe the changes in cell skeleton and cell morphology (Figure 1b). The F-actin filaments in the untreated group were well organized in bundles that stretched between the cell surface and the cytoplasm, and the cells were spindle shaped. Compared with the untreated group, the filaments in the treated groups by COM-50 nm and COD-50 nm appeared shrunken and disorganized. Moreover, the cells obviously shrank, presenting obvious characteristics of apoptotic cells. Although most cells in the treated groups by COM-10 *μ*m and COD-10 *μ*m changed slightly, some cells became swollen and filaments became vague, presenting obvious characteristics of necrotic cells.

### Lysosomal integrity and distribution

Besides the morphological observation, biochemical methods for assessing cell death were essential for the in-depth study of intracellular parameters.^13^ Acridine orange (AO), a metachromatic fluoro-probe, is a lysosomotropic component that can accumulate in lysosomes by proton trapping. AO accumulation produces a change in the fluorescence emission from green in the cytoplasm to red in the lysosomes. The lysosomes in normal and early apoptotic cells retained their intact structure and emitted stronger red fluorescence, whereas the lysosomes in necrotic cells were ruptured and emitted weaker red fluorescence.^21^

**Figure 2:**
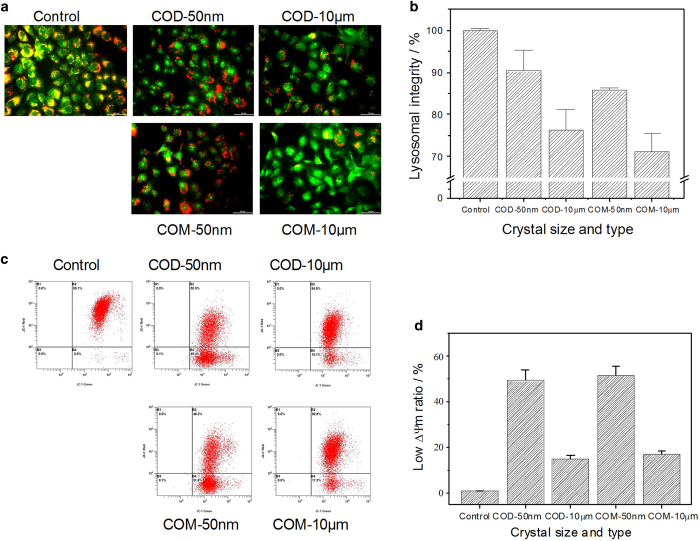
Lysosomal integrity observation and mitochondrial membrane potential detection after exposing to 200 *μ*g/ml nano-/micron-sized COM and COD crystals for 6 h. (**a**) Fluorescence microscope observation and (**b**) quantitative analysis of lysosomal integrity in Vero cells. Red fluorescence represented unbroken lysosomes. (**c**) Flow cytometric data of mitochondrial membrane potential (Δ*ψ*m) and (**d)** quantitative histogram of Δ*ψ*m.

The extent of lysosomal membrane destabilization in Vero cells was visualized under fluorescence microscopy (Figure 2a), which was then quantitatively analyzed using a microplate reader (Figure 2b). In the control group, the AO-loaded lysosomes were uniformly distributed inside the Vero cells. The emitted red fluorescence of lysosomes merged with the green fluorescence of the cytoplasm, thereby presenting much orange fluorescence. The cells and the cytoplasm remarkably shrank in the COM-50 nm and COD-50 nm treatment groups, presenting obvious apoptotic features. Most lysosomes kept their integrity in nano-sized crystal-treated groups, and the unbroken lysosomes were 85.70±0.75% and 90.31±5.00% in COM-50 nm- and COD-50 nm-treated cells, respectively. However, COM-10 *μ*m and COD-10 *μ*m crystals severely damaged lysosomes and disturbed AO inside cells. The integrity was 71.16%±4.29% and 76.24%±4.87% in COM-10 *μ*m- and COD-10 *μ*m-treated cells, respectively. The ability of the four kinds of crystals to cause lysosome injury was COM-10 *μ*m>COD-10 *μ*m>>COM-50 nm>COD-50 nm. Lysosomal rupture is an important characteristic of necrosis,^22^ so micron-sized crystals were more likely to lead to necrotic cell death in this study.

### Decrease in mitochondrial membrane potential (Δ*ψ*m)

Apoptosis and necrosis are often preceded by mitochondrial dysfunction, in particular, a loss of mitochondrial membrane potential (Δ*ψ*m). Mitochondria have high Δ*ψ*m potential under normal circumstances, and would become depolarized after suffering injury. The Δ*ψ*m of Vero cells after exposure to nano-/micron-sized COM and COD crystals is shown in Figures 2c and d. The JC-1 dye was used to determine the effects of nano-/micron-sized COM and COD exposure on the Δ*ψ*m in Vero cells. Nano-sized COM and COD crystals caused significant decrease in Δ*ψ*m than in control cells, whereas micron-sized crystals caused only slight decrease in Δ*ψ*m. Nano-sized crystals caused much more serious injury to mitochondria than the micron-sized crystals. Meanwhile, the decrease in Δ*ψ*m caused by COM was slightly higher than COD with the same size.

### Fluorescence microscopy of apoptosis and necrosis

**Figure 3:**
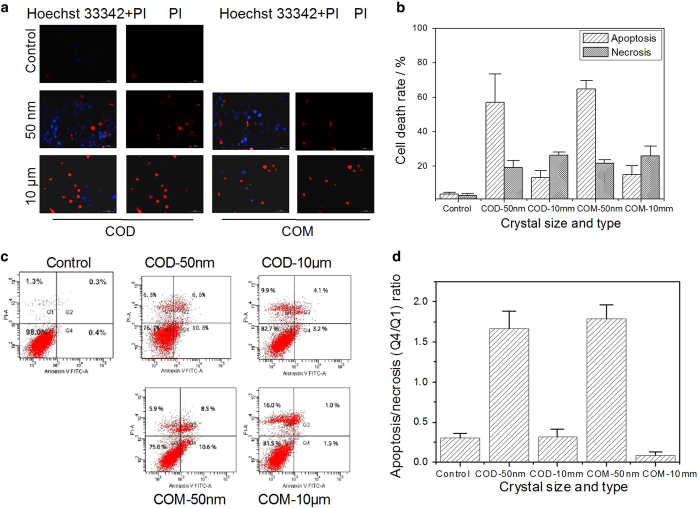
Apoptosis and necrosis assay after exposing to 200 *μ*g/ml nano-/micron-sized COM and COD crystals for 6 h. (**a**) Fluorescence microscope observation of apoptosis and necrosis of Vero cells by Hoechst 33342 (blue) and propidium iodide (red) staining. (**b**) Quantitative results of apoptosis and necrosis by analyzing fluorescence intensity. (**c**) Flow cytometric data of apoptosis and necrosis in Vero cells by annexin V/PI double staining. (**d**) Apoptosis/necrosis (Q4/Q1) ratio. Quadrants Q1, Q2, Q3, and Q4 denote the ratio of necrotic cells, necrotic and/or late apoptotic cells, normal cells, and early apoptotic cells, respectively.

Hoechst 33342 can penetrate through normal and apoptotic cell membranes and bind to intracellular DNA and emit blue fluorescence, but the fluorescence intensity in apoptotic cells was obviously higher than that in normal cells. Propidium iodide (PI) cannot penetrate through normal cell membranes but can pass through necrotic cell membranes and bind to DNA in the nucleus and emit red fluorescence. Thus, we double stained cells with Hoechst 33342 as a measure of apoptosis and with PI as an indicator of membrane integrity and thus necrosis (Figures 3a and b).

The number of stained cells (blue and red) in the control group was low. The number of stained cells (blue and red) and fluorescence intensity in the COM-50 nm and COD-50 nm treatment groups were both greater than those in the COM-10 *μ*m and COD-10 *μ*m treatment groups. The number of cells stained by Hoechst 33342 (blue) was far more than that stained by PI (red) in the nano-sized treatment groups, whereas red fluorescence was obviously stronger than blue fluorescence in the micron-sized treatment groups. Therefore, nano-/micron-sized COM and COD crystals could simultaneously cause apoptosis and necrosis, but nano-sized crystals primarily caused apoptotic cell death, whereas micron-sized crystals primarily caused necrotic cell death. Meanwhile, nano-sized crystals caused more cell death than micron-sized crystals.

### Flow cytometry of apoptosis and necrosis

To further assess the nature of cell death induced by various sizes of COM and COD crystals, we performed flow cytometry to quantitate apoptotic and necrotic cells using annexin V/PI double staining (Figure 3c). Annexin V staining was applied to reveal the surface exposure of PS (apoptosis), and PI staining was applied to reveal the loss of plasma membrane integrity (necrosis).

The four quadrants, namely, Q1, Q2, Q3, and Q4, denote the ratio of necrotic cells, late apoptotic cells and/or necrotic cells, normal cells, and early-stage apoptotic cells, respectively. Necrotic cells were difficult to distinguish from late apoptotic cells when annexin V/PI double staining was performed in Q2 alone. Meanwhile, quadrant Q1 may be also a mixed region containing necrotic cells and some of dead cells by mechanical injury or some of nuclei from broken cells. During the experimental process, we carefully treated the cell samples to avoid mechanical injury, and the number of nuclei from broken cells should be much less than necrotic cells. Meanwhile, extensive researches regarded that quadrant Q1 denoted the number of necrotic cells.^23–25^ Thus, we selected Q4 (apoptotic cells) and Q1 (necrotic cells) to compare the number of apoptotic and necrotic cells to some extent (Figure 3d). Nano-sized crystals (COM-50 nm and COD-50 nm) mainly caused apoptotic cell death, and the apoptosis/necrosis (Q4/Q1) ratio was 1.79 and 1.67, respectively. Micron-sized crystals (COM-10 *μ*m and COM-10 *μ*m) mainly caused necrotic cell death, and the Q4/Q1 ratio was only 0.09 and 0.32, respectively.

### Malondialdehyde generation during apoptosis and necrosis

Cell injury could lead to series of changes of cellular biochemical parameters, of which the change of the released amount of malondialdehyde (MDA) can reflect the degree of lipid peroxidation in biomembrane, and it indirectly reflects the degree of cell injury.

**Figure 4:**
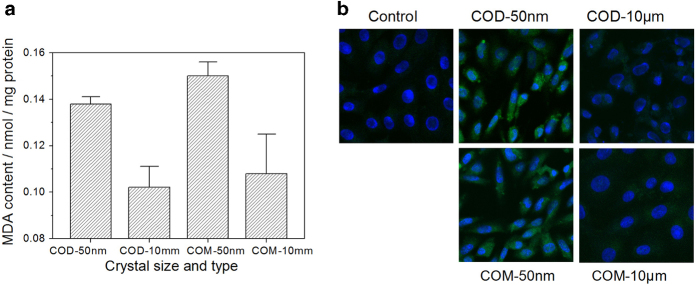
MDA release amount and OPN expression after treatment with 200 *μ*g/ml nano-/micron-sized COM and COD crystals for 6 h. (**a)** MDA release amount in Vero cells after treatment by COM and COD crystals. **(b**) OPN expression observation by fluorescence staining.

The changes in the generated MDA amount in Vero cells are shown in Figure 4a. The MDA content in the nano-sized crystal-treated groups was much greater than that in the micron-sized crystal-treated groups. COM crystals caused more serious injury to cells than the same-sized COD crystals. The released amount of MDA was consistent with the degree of Vero cell injury.

### OPN expression during apoptosis and necrosis

Apoptosis and necrosis could promote OPN expression. OPN is an important adhesion molecule on the cell surface, which can be upregulated after cell injury.^26^ Thus, we used a fluorescent molecular marker to observe the expression of OPN by confocal laser scanning microscopy. OPN expression in Vero cells after treatment with nano-/micron-sized crystals is shown in Figure 4b. Compared with the control group in which minimal OPN expression was observed, all the crystal-treated groups showed obvious green fluorescence. The nano-sized crystal-treated groups induced much higher OPN expression levels than the micron-sized crystal-treated groups, and COM caused more OPN expression than the same-sized COD. In addition, we also observed that the cell nucleus in the nano-sized crystal-treated group was much smaller than that of the control groups and micron-sized crystal-treated groups, presenting obvious apoptotic features.

## Discussion

Studies *in vitro* ^5,26–28^ and *in vivo* ^29–31^ have demonstrated that CaOx crystals are toxic for renal epithelial cells, producing injury and death of renal cells, but the cell death mode induced by CaOx crystals varied in different studies. The recognition that apoptotic and/or necrotic cell death can have an important role in defining the mode of cell death, produced by nano-/micron-sized COM and COD crystals. Many researches on a number of other toxins have suggested that the mode of cell death may vary, depending on the severity of the insult, with apoptosis occurring in response to injurious stimuli of lesser magnitude and necrosis occurring in response to more severe injury.^14,32,33^ The results in this study demonstrated that exposure to nano-/micron-sized COM and COD crystals triggered both apoptotic and necrotic cell death in renal epithelial cell lines. However, nano-sized crystals primarily induced cell apoptosis, whereas micron-sized crystals primarily induced cell necrosis.

### Nano-sized COM and COD crystals primarily induce apoptotic cell death

**Figure 5:**
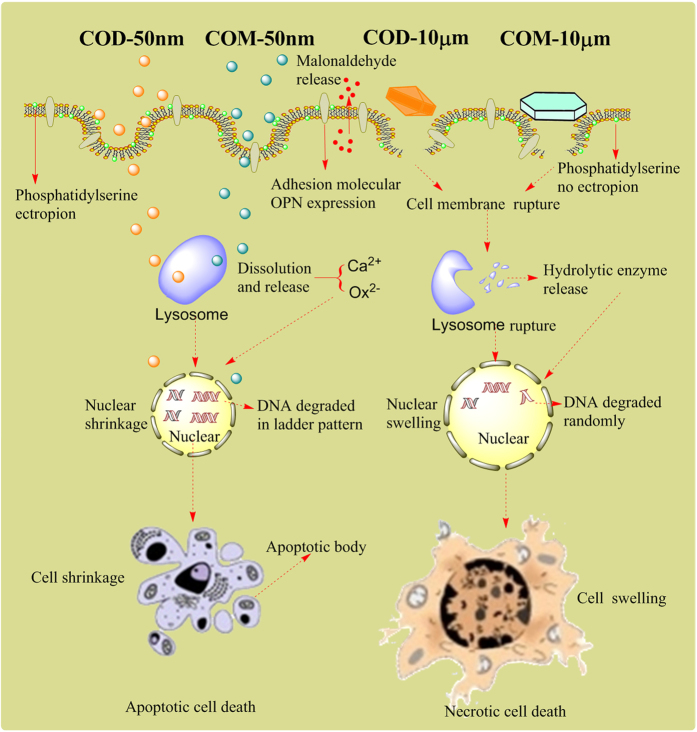
Suggested schematic illustration of cell death mode caused by nano-/micron-sized COM and COD crystals exposure. COD-50 nm and COM-50 nm primary caused apoptotic cell death due to their small size effect and uniform and moderate injuries. COD-10 *μ*m and COM-10 *μ*m primary caused necrotic cell death due to their large size and local acute injuries.

Given the small size, large specific surface area, and numerous exposed surface active sites of nano-sized crystals (COM-50 nm and COD-50 nm), they showed higher cytotoxicity than micron-sized crystals. In the nano-sized crystal-treated groups, cells shrank and lost microvilli and cell junction. Moreover, actin distribution was disrupted (Figure 1b). These findings were obvious characteristics of apoptotic cells. The cell death mechanism induced by nano-sized COM and COD is summarized in the schematic in Figure 5.

Nano-sized crystals are more likely to be internalized by cells than micron-sized crystals. These internalized nano-sized COM and COD crystals were distributed in the cytoplasm, and some of the crystals were transferred into lysosomes via vesicular transport. Lysosomes are important organelles for digesting exogenous substances; intracellular lysosomes contain various acid hydrolases, and the pH in lysosomes is approximately 4.5.^34^ The internalized nano-sized COM and COD crystals could be degraded readily in lysosomes to release calcium and oxalate ions.^35^ Meanwhile, internalized nano-sized crystals may increase the lysosomal membrane permeability in varying degrees. A prevalent assumption is that the reparable damage of lysosomes can initiate apoptosis, and a sudden massive destruction of lysosomes leads to necrosis.^36^ The concentration of nano-sized crystals used in this study was lower and these crystals were evenly distributed on the cell surface, hence they induced mild injury instead of partial acute injury. Intracellular lysosomes maintained good integrity in the nano-sized crystal-treated groups (Figure 2a) and did not show obvious differences compared with control cells. However, the cells shrank, presenting obvious apoptosis characteristics. Furthermore, nano-sized crystals caused much more serious injury to mitochondria and caused more significantly decrease in Δ*ψ*m than micron-sized crystals (Figure 2c). Nano-sized crystals were easily to be internalized by Vero cells due to their small size; these internalized nano-sized crystals can directly affect the mitochondria, thereby disturbing the metabolic balance, and lead to mitochondrial membrane depolarization and Δ*ψ*m decrease.

In addition, apoptotic cells maintained their plasma membrane integrity, but PS was translocated on the cell membrane, which could be stained by annexin V (Figure 3c). Clusters of negatively charged head groups of PS attract calcium and act as sites for the attachment of calcific crystals to the cell surfaces.^37^ Fluorescence microscopy and flow cytometry were used to analyze cell apoptosis and necrosis. Nano-sized crystals were more likely to induce cell apoptosis and a small fraction of necrosis (Figures 3a and c). This finding was similar to the toxicity manner of oxalate in renal epithelial cells, which can cause both apoptotic and necrotic cell death.^14^ This phenomenon may be the reason why nano-sized crystals were easily dissolved by lysosomes to release oxalate ions. The internalized crystals could not only be captured by lysosomes but also entered into the nucleus through the nuclear pores, leading to the cleavage of DNA into internucleosomal fragments of 180 bp and multiples,^38^ which is an important characteristic of apoptotic cell death. In this study, nano-sized COM and COD crystals caused relatively low apoptosis rate (only about 10%; Figure 3c) and simultaneously induced a small fraction of necrosis. Therefore, DNA ladder experiments were unsuitable for distinguishing cell apoptosis and necrosis in this study.

Cell apoptosis and a small fraction of necrosis induced by nano-sized COM and COD crystals were accompanied with lipid peroxidation, which released lipid peroxidation products, such as MDA (Figure 4a), changed cell membrane fluidity and permeability, and affected the cell structure and function. Nano-sized crystals had larger specific surface area and more active sites than micron-sized crystals under the same concentrations; these active sites could capture oxygen molecules and produce superoxide radical.^39^ Therefore, nano-sized crystals caused a greater degree of lipid peroxidation and generated more MDA. In addition, injured cells also expressed a large amount of the adhesion molecule OPN (Figure 4b). Previous studies showed that immobilized OPN on the cell surface promotes crystal attachment. Pretreatment of MDCK cells with polyclonal antibodies against OPN, as well as transfection of NRK52E cells with antisense OPN cDNA, could reduce adhesion of CaOx crystals.^40^ Therefore, higher OPN expression levels will aggravate crystal adhesion on the cell surface. The adhesion force between COM crystals and the expressed adhesion molecule (OPN) on the cell surface of injured cells should be higher than that between COD crystals and OPN.^41^ Therefore, COM crystals induced higher OPN expression levels and easily adhered on the cell surface, thereby causing serious cellular damage.

### Micron-sized COM and COD crystals primarily induce necrotic cell death

Micron-sized crystals (COM-10 *μ*m and COD-10 *μ*m) were difficult to be internalized by cells and mainly adhered on the cell surface because of their large size. These adhered micron-sized crystals could directly lead to cell membrane damage, which further induced an intracellular response. Micron-sized crystals caused cell swelling, cytoskeleton abnormalities, and cell membrane and lysosome rupture, presenting obvious characteristics of necrotic cells. Meanwhile, micron-sized crystals also could cause mitochondria depolarization, but the decrease of Δ*ψ*m in micron-sized crystal-treated groups was much lower than nano-sized crystal-treated groups. Micron-sized crystals used in this study were too large to be internalized by Vero cells, these crystals mainly adhered to cell surface and could not directly contact with intracellular mitochondria, and thus causing slightly decrease of Δ*ψ*m. These results implied that apoptosis process may be more easily to cause mitochondria damage than necrosis process. The cell death mechanism induced by micron-sized COM and COD is also summarized in the schematic illustrated in Figure 5.

The number of micron-sized crystals was much less than that of nano-sized crystals under the same concentration. Micron-sized crystals on cells presented a nonhomogeneous distribution, which caused uneven injury of the cell membrane and local strong physical stress, resulting in necrotic cell death. Necrotic cells released inflammatory factors^42^ and led to cytomembrane rupture based on the findings of PI staining in Figures 3a and c,^8^ thereby further deteriorating the external cell environment and causing further cell necrosis. Cell membrane rupture can cause an imbalance in cell osmotic pressure and lead to the sudden massive destruction of lysosomes (Figure 2a) accompanied with hydrolytic enzyme release, which is an important factor in necrotic cell death.^36,43^ Both fluorescence microscopy and flow cytometry confirmed that micron-sized crystals induced cell necrosis compared with nano-sized crystals. Meanwhile, sudden massive release of hydrolytic enzyme could lead to the random degradation of chromatin DNA, resulting in necrotic cell death.^25^

Cell necrosis and a small fraction of apoptosis induced by micron-sized COM and COD crystals were also accompanied with lipid peroxidation and OPN expression, but the released MDA and expressed OPN were much less than in the nano-sized crystal-treated group (Figures 4a and b). The degree of lipid peroxidation was related to the ability of particles to capture oxygen molecules. The exposed active sites of micron-sized crystals were much less than those of nano-sized crystals, significantly weakening their capacity for cell damage. Cell apoptosis induced by nano-sized COM and COD was accompanied with PS ectropion, whereas necrosis induced by micron-sized crystals was not. This exposed negatively charged PS acted as a binding site of urine microcrystalline on the cell surface and increased the adhesion and aggregation of microcrystallines, thereby increasing the risk of stone formation.^44^ Therefore, compared with necrosis, apoptosis may be more likely to induce stone formation.

Nano-sized COM and COD crystals presented higher cytotoxicity than micron-sized crystals under the same concentration, but cell damage caused by nano-sized COM and COD crystals was more moderate and homogeneous. Nano-sized crystals did not easily cause local acute injuries, and they were more likely to lead to apoptotic cell death. Micron-sized crystals caused less injury than nano-sized crystals, but micron-sized crystals could induce strong physical stress and lead to local acute injuries on the cell surface because of their large size, which were more likely to lead to necrotic cell death. Therefore, renal epithelial cells could be injured by exposed crystals and undergo apoptosis and/or necrosis, initiating a cascade of events, such as lipid peroxidation and OPN expression, leading to further crystallization, crystal retention, and development of stone nidi.

## Conclusion

CaOx with different sizes and crystal phase have been used as model systems to investigate the influence of these two factors on cytotoxicity and cell death mode. Cell death mode was closely related to crystal size. Nano-/micron-sized COM and COD crystals exposure triggers both apoptotic cell death and necrotic cell death. But nano-sized crystals primary caused more homogeneous damage and induced apoptotic cell death due to their small size and uniform distribution on the cell surface, causing cell shrinkage, mitochondria depolarization, PS ectropion, and nuclear shrinkage. However, micron-sized crystals could cause local acute injuries of cell surface and induce necrotic cell death due to their large size and inhomogeneous distribution, causing cell swelling, and cell membrane and intracellular lysosome rupture. The cytotoxicity of nano-sized crystals was higher than that of micron-sized crystals under the same concentration, and the cytotoxicity of COM crystals was higher than that of the same-sized COD crystals. The study of recognizing apoptosis and necrosis induced by nano-/micron-sized crystals will be conducive to reveal the influence of the physicochemical properties of exogenous materials on the mode of cell death.

## Materials and Methods

### Reagents and apparatus

African green monkey renal epithelial (Vero) cells were purchased from Shanghai Cell Bank, Chinese Academy of Sciences (Shanghai, China). Dulbecco’s modified eagle medium (DMEM), fetal bovine serum, penicillin, and streptomycin were obtained from HyClone Biochemical Products Co., Ltd. (Logan, UT, USA). Trypsin and phosphate-buffered saline (PBS) were purchased from Beijing Pubo Biotechnology Co., Ltd. (Beijing, China). Acridine orange were purchased from Sigma-Aldrich (St. Louis, MO, USA). FITC-conjugated phalloidin, 4′, 6-diamidino-2-phenylindole (DAPI), anti-fade fluorescence mounting medium, bovine serum albumin (BSA), 5,5′,6,6′-tetrachloro-1,1′,3,3′-tetraethyl-imidacarbocyanine iodide (JC-1), Annexin V-FITC and Hoechst 33342/PI were all purchased from Shanghai Beyotime Bio-Tech Co., Ltd. (Shanghai, China). The first antibody of OPN and FITC secondary antibody were purchased from Santa Cruz (Dallas, TX, USA). MDA assay kit was obtained from Nanjing Jiancheng Bioengineering Institute. Cell culture plates were purchased from Wuxi Nest Bio-Tech Co., Ltd. (Wuxi, China).

The apparatus used in this paper were as following: X-L type environmental SEM (Philips, Eindhoven, Netherlands); confocal laser scanning microscopy (LSM510 Meta Duo Scan, Zeiss, Jena, Germany); microplate reader (Safire2, Tecan, Männedorf, Switzerland); flow cytometer (FACS Aria, BD Corporation, Franklin Lakes, NJ, USA); fluorescence microscope (IX51, Olympus, Tokyo, Japan); TECNAI-10 TEM (Philips) running at accelerating voltage of 100 kV; and Fourier transform infrared spectrometry (Nicolet Company, Madison, WI, USA).

### Experimental methods

#### Preparation and characterization of nano-/micron-sized COM and COD crystals

According to our recent paper,^20^ COM and COD crystals with the sizes of about 50 nm and 10 *μ*m were prepared by changing the concentration of reactants (CaCl_2_ and Na_2_Ox), reaction temperature, solvent, and stirring speed. The size and crystalline phase of prepared crystals were characterized by SEM, TEM, XRD, and FI-IR.

For the preparation of different-sized CaOx crystal suspension, a certain amount of COM or COD crystals were UV sterilized for 40 min and dispersed in serum-free DMEM culture medium at a concentration of 200 *μ*g/ml.

#### Cell culture

Vero cells were cultured in a DMEM culture medium containing 10% fetal bovine serum, 100 U/ml penicillin–100 *μ*g/ml streptomycin antibiotics with pH 7.4 at 37 °C in a 5% CO_2_ humidified environment. Upon reaching 80–90% confluent monolayer, cells were blown gently after trypsin digestion to form cell suspension for the following cell experiment.

#### Cytoskeleton observation

About 1 ml of cell suspension with a cell concentration of 1×10^5^ cells/ml was inoculated per well in 12-well plates for 12 h. The nano-/micron-sized crystals with a concentration of 200 *μ*g/ml were incubated with subconfluent cultures at 37 °C. After 6 h of incubation, the supernatant was aspirated, and the cells were washed twice with PBS. Then cells were fixed for 30 min with paraformaldehyde (4%) in PBS, followed by three rounds of membrane permeabilization with 0.1% Triton X-100 in PBS at room temperature for 5 min. The slides were incubated for 60 min with 200 *μ*l of FITC-conjugated phalloidin and washed twice with 0.1% Triton X-100. DAPI staining solution was then added to the cells and incubated for 5 min. The cells were again washed three times with PBS for 5 min. Finally, the prepared samples were mounted with anti-fade fluorescence mounting medium and observed by confocal laser scanning microscopy.

#### Lysosomal distribution and integrity

The cell suspension (1 ml) with a cell concentration of 1×10^5^ cells/ml was grown to sub-confluence in 12-well plates with coverslips for 24 h. The cells were then loaded with 5 *μ*g/ml AO in DMEM for 15 min. Subsequently, the culture media were discarded. The cells were rinsed with PBS and then incubated in serum-free culture media with 200 *μ*g/ml nano-/micron-sized COM and COD crystals for 6 h. The cells were washed with PBS before fluorescence measurements with excitation at 485 nm and emission at 530 (green cytoplasmic AO) and 620 nm (red lysosomal AO). Normal lysosomal integrity=(total red fluorescence intensity)/(total green fluorescence intensity). Lysosomal integrity=(total red fluorescence intensity)/((total green fluorescence intensity)×(normal lysosomal integrity)).

#### Measurement of mitochondrial membrane potential (Δ*ψ*m)

The cell suspension (2 ml) with a concentration of 1×10^5^ cells/ml was inoculated per well in 6-well plates for 12 h. After 6 h of incubation with nano-/micron-sized COD and COM crystals at the concentration of 200 *μ*g/ml, the supernatant was aspirated and the cells were washed twice with PBS and digested with 0.25% trypsin. The cells were suspended by pipetting, followed by centrifugation (1000 r.p.m., 5 min). The supernatant was aspirated and the cells were washed with PBS and centrifuged again to obtain a cell pellet. The cells were resuspended by adding and thoroughly mixing 500 *μ*l of PBS in a microcentrifuge tube. Finally, the samples were stained with JC-1 and then analyzed.

#### Fluorescence microscopy observation of cell apoptosis and necrosis

The density of seeded cells and experimental grouping were the same as those in lysosomal distribution and integrity assay. Afterward, the culture media were discarded and the cells were rinsed with PBS and then incubated in serum-free culture media with COM and COD crystals for 6 h. Then the cells were washed with PBS for three times and stained using 5 *μ*l of Hoechst 33342/PI for 20 min at 4 °C. The Hoechst 33342 exhibited blue fluorescence and PI exhibited red fluorescence.

#### Flow cytometer quantitative analysis of cell apoptosis and necrosis

The density of seeded cells and experimental grouping were the same as those in lysosomal distribution and integrity assay. The collected cells stained using Annexin V-FITC/PI cell death assay kit according to the manufacturer’s instructions. About 1.5×10^5^ cells were collected and washed with PBS (centrifuged at 1000 r.p.m. for 5 min). The cells were resuspended in 200 *μ*l binding buffer. Afterward, 5 *μ*l Annexin V-FITC was added and then incubated in darkness at room temperature for 10 min. The cells were again resuspended in 200 *μ*l binding buffer and stained with 5 *μ*l PI. The prepared cells were then analyzed using a flow cytometer.

#### OPN expression assay

The density of seeded cells and experimental grouping were the same as those in lysosomal distribution and integrity assay. Afterward, cells were then fixed with 4% paraformaldehyde for 10 min. Then the cells were washed with PBS three times for 3 min each. The cells were incubated in sheep serum for 20 min, and afterward the first OPN antibody (1:100) (Santa Cruz) was mixed into the samples and incubated at 4 °C overnight. The cells were then washed with PBS three times for 5 min each. Afterward, FITC secondary antibody (1:100) (CA, USA) was added to the cells in the dark and incubated at 37 °C for 0.5 h. The cells were again washed with PBS three times for 5 min each. Finally, the cells were stained and sealed with DAPI. Fluorescence was observed using a laser confocal fluorescence microscope. OPN and cell nuclei were stained green and blue, respectively.

#### MDA detection

The cell suspension was inoculated in 24-well plates with a cell concentration of 1×10^5^ cells/ml and 500 *μ*l per well, and was incubated for 24 h in an incubator at 37 °C. The cells were divided into five groups, of which one was the control group and the others were crystal-treated groups, with each well repeatedly measured three times. The injured groups were exposed to serum-free culture medium with nano-/micron-sized crystals, 200 *μ*l per well, and incubated for 6 h. Subsequently, the liquid supernatant was absorbed for MDA measurement conducted strictly according to the instructions provided with the kit.

#### Statistical analysis

The experimental results were analyzed statistically using SPSS 13.0 software (SPSS Inc., Chicago, IL, USA) and expressed as mean±S.D. from three independent experiments.
